# A Novel Stimuli‐Responsive Injectable Antibacterial Hydrogel to Achieve Synergetic Photothermal/Gene‐Targeted Therapy towards Uveal Melanoma

**DOI:** 10.1002/advs.202004721

**Published:** 2021-07-31

**Authors:** Shaoyun Wang, Baohui Chen, Liping Ouyang, Donghui Wang, Ji Tan, Yuqin Qiao, Shengfang Ge, Jing Ruan, Ai Zhuang, Xuanyong Liu, Renbing Jia

**Affiliations:** ^1^ Department of Ophthalmology Ninth People's Hospital Shanghai JiaoTong University School of Medicine Shanghai 200025 P. R. China; ^2^ State Key Laboratory of High Performance Ceramics and Superfine Microstructure Shanghai Institute of Ceramics Chinese Academy of Sciences Shanghai 200050 P. R. China; ^3^ Shanghai Key Laboratory of Orbital Diseases and Ocular Oncology Shanghai 200025 P. R. China; ^4^ Center of Materials Science and Optoelectronics Engineering University of Chinese Academy of Sciences Beijing 100049 P. R. China; ^5^ Cixi Center of Biomaterial Surface Engineering Shanghai Institute of Ceramics Chinese Academy of Sciences Ningbo 315300 P. R. China; ^6^ School of Chemistry and Materials Science Hangzhou Institute for Advanced Study University of Chinese Academy of Sciences Hangzhou 310024 P. R. China

**Keywords:** antibacterial activity, gene‐targeted therapy, injectable hydrogel, intelligent release, photothermal therapy, uveal melanoma

## Abstract

Uveal melanoma (UM) is the most prevalent primary intraocular malignant tumor with a high lethal rate. Patients who undergo conventional enucleation treatments consistently suffer permanent blindness, facial defects, and mental disorders, therefore, novel therapeutic modalities are urgently required. Herein, an injectable and stimuli‐responsive drug delivery antibacterial hydrogel (CP@Au@DC_AC50) is constructed via a facile grinding method that is inspired by the preparation process of traditional Chinese medicine. The incorporation of gold nanorods can enhance the mechanical strength of the hydrogel and realize photothermal therapy (PTT) and thermosensitive gel‐sol transformation to release the gene‐targeted drug DC_AC50 on demand in response to low‐density near‐infrared (NIR) light. The orthotopic model of UM is built successfully and indicates the excellent efficiency of CP@Au@DC_AC50 in killing tumors without damage to normal tissue because of its synergistic mild temperature PTT and gene‐targeted therapy. Moreover, the eyeball infection model reveals the remarkable antibacterial properties of the hydrogel which can prevent endophthalmitis in the eyeball. There is negligible difference between the CP@Au@DC_AC50+NIR group and normal group. This NIR light‐triggered gene‐targeted therapy/PTT/antibacterial treatment pattern provides a promising strategy for building multifunctional therapeutic platform against intraocular tumors and exhibits great potential for the clinical treatment of UM.

## Graphical abstract

Inspired by the grinding method in traditional Chinese medicine preparation, the injectable antibacterial self‐assembling nanofiber CP@Au@DC_AC50 hydrogels are first constructed. NIR light‐triggered drug release/photothermal/gene‐targeted therapy of CP@Au@DC_AC50 exhibits brilliant synergistic antitumor and antibacterial efficiency without damage to intraocular tissue in vitro and in vivo. The gene‐targeted therapy/PTT/antibacterial platform has potential for future application in ocular tumor therapy.

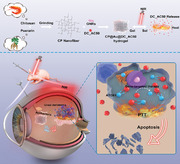

## 1 Introduction

Uveal melanoma (UM) is the most common primary intraocular malignant tumor in adults.^[^ ^1^ ^]^ With tumor progression, patients often experience symptoms such as exophthalmos, soft periocular tissue edema, intraocular hemorrhage, and eyeball mobility disturbance.^[^ ^2^, ^3^ ^]^ Eventually, tumor cells break through the sclera with the development of systemic metastasis. Although enucleation is the most reliable treatment for patients, the major side effects of blindness and severe mental disorders caused by permanent facial disfigurements limit its widespread application.^[^ ^4^ ^]^ These limitations lead to a quest for the development of novel treatments against UM. As a standby for cancer therapy, chemotherapy has been applied in UM.^[^ ^5^ ^]^ However, clinical research has shown that single‐agent or combined systemic chemotherapy is often associated with poor prognosis, mainly caused by two factors. The first is that systemic chemotherapy is always accompanied by low drug concentrations in the eyeball because of the blood‐ocular barrier, and the second is chemotherapy resistance.^[^ ^5^, ^6^ ^]^ Therefore, the key to UM treatment lies in achieving higher intraocular drug concentrations and finding sensitive drugs to replace traditional chemotherapy drugs.

Eyeball injection is the most effective way to enhance intraocular drug accumulation.^[^ ^7^, ^8^ ^]^ However, multiple intraocular injections are not only inconvenient to daily operations but also increase the incidence of complications, including edema, bleeding, and eyeball deformation.^[^ ^9^ ^]^ This gives rise to the need to develop a safe biological carrier to realize a single injection for the long‐term and on‐demand release of encapsulated drugs. Injectable hydrogels based on natural polymers have emerged as promising platforms for biomedical applications, including drug delivery,^[^ ^10^, ^11^, ^12^ ^]^ antibacterial infection,^[^ ^13^, ^14^ ^]^ orthopedic repair^[^ ^15^, ^16^, ^17^ ^]^ and irregularly shaped wound healing^[^ ^18^, ^19^, ^20^ ^]^ due to their biocompatibility, flexible mechanical controllability, and environmental responsiveness.^[^ ^21^, ^22^, ^23^ ^]^ Localized therapy with injectable hydrogels can increase the amount of drug that reaches the tumor site in eyeballs and decrease systemic side effects.^[^ ^24^ ^]^ Although injectable hydrogels have been the subject of extensive research in recent years, designing drug delivery hydrogels that could be applied to intraocular tumor treatment is still a great challenge, due to the unique microenvironment of the eyeballs. An ideal drug‐loading hydrogel platform for eyeball injection treatment against UM should possess the following characteristics: 1) suitable mechanical strength after injection to resist intraocular pressure of 12 − 21 ± 2 mmHg (≈1.3–3.0 kPa), low strength would lead to gel rupture whereas excessive strength results in discomfort, disfigurement, and functional impairments; 2) on demand release of drugs. Although many tumor environment responsive hydrogels have been reported, these kinds of hydrogels could hardly realize sustained, long‐term, and smart controlled release in vitro and in vivo; 3) extremely high biosafety and biocompatibility. Chemical crosslink hydrogels and traditional chemotherapeutic drugs might cause damage to normal tissue in eyeballs; 4) antibacterial ability. As an invasive treatment, eyeball injection could cause endophthalmitis which is one of the most devastating eyeball infections and leads to severe necrosis and cell death in various internal structures and inreversible blindness.^[^ ^25^, ^26^ ^]^ Moreover, the latest research shows that bacteria play an important role in tumorigenesis, and the rate of intratumor bacteria in patients is ≈14.3% to ≈60%.^[^ ^27^, ^28^, ^29^, ^30^ ^]^ To avoid endophthalmitis caused by eyeball injection and effectively kill bacteria harbored in the primary tumor site, biological carriers should exhibit excellent antibacterial properties. Previously reported drug‐loading hydrogels may meet one or two of the above requirements, but could hardly satisfy all the demands.

**Scheme 1:**
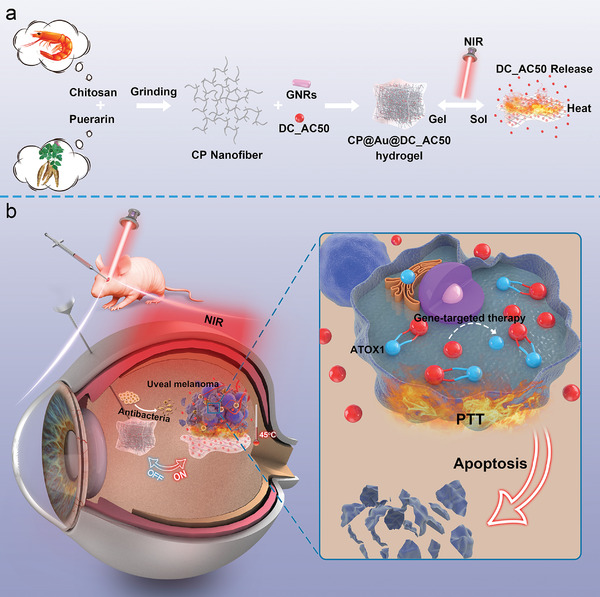
Scheme 1. Schematic illustration of (a) the construction and NIR light‐triggered gel‐sol transition and (b) the synergistic gene‐targeted therapy/low‐temperature photothermal/anti‐infection abilities of the CP@Au@DC_AC50 hydrogel for UM treatment.

In this study, an injectable gold nanorods (GNRs) incorporated chitosan@puerarin (CP) hydrogel was constructed as the drug carrier and the gene‐targeted drug DC_AC50 was selected to replace traditional drugs. Traditional Chinese medicine (TCM) has been widely used for prevention and treatment of diseases for 2000 years.^[^ ^31^ ^]^ Puerarin, an active and main constituent isolated from *Pueraria* (a famous TCM), can spontaneous assemble into a nanofiber hydrogel under a rapid heating–cooling process.^[^ ^32^, ^33^ ^]^ However, a single component of puerarin hydrogel cannot be directly injected owing to the rigid structure of nanofiber. Derived from deacetylation of the natural polymer chitin which is the main component of shrimp and crabs, chitosan has exhibited excellent biocompatibility and biodegradability as well as antibacterial abilities.^[^ ^34^, ^35^ ^]^ Inspired by the traditional Chinese grinding method in TCM preparation, a simple one‐step method of grinding was adopted to synthesize the injectable antibacterial supramolecular self‐assembling nanofiber CP hydrogels with the incorporation of flexible polymer chains of chitosan.^[^ ^32^, ^33^ ^]^ On the one hand, no cytotoxic component was introduced in the drug loading system, guaranteeing its high biocompatibility. On the other hand, the incorporation of gold nanorods into the drug‐loaded CP gel could achieve two goals, first, the mechanical properties of the CP@Au gel could be regulated by adjusting the GNRs doping amounts to satisfy the special mechanical requirement of eyeballs, and second, taking advantage of the excellent photothermal conversion effect of GNRs, NIR light can serve as a trigger for the gel‐sol transformation to realize the controlled release of drugs and mild temperature photothermal therapy.^[^ ^36^ ^]^ In addition to mechanically adjustable, smart drug release, and antibacterial injectable hydrogel carriers, sensitive and safe drugs are still needed. Instead of conventional chemotherapy, gene‐targeted therapy utilizing specific small‐molecule inhibitors is suitable for the treatment of UM because of the remarkable ability to achieve rapid, efficient, and specific tumor killing without damage to normal tissue.^[^ ^37^, ^38^ ^]^ Based on differentially expressed genes identified by genome‐wide RNA sequencing, we found that the antioxidant 1 Copper Chaperone (ATOX1) was upregulated in UM cells. It has been reported that DC_AC50, a small‐molecule inhibitor of ATOX1, possesses excellent targeted antitumor ability, therefore, DC_AC50 was selected for UM treatment platform construction.^[^ ^39^ ^]^ Therefore, the developed multifunctional bioactive injectable nanofiber hydrogel CP@Au@DC_AC50 possessed shear‐thinning, self‐healing, photothermal conversion, effective drug loading, and controlled release as well as antibacterial capacity against eyeball injection for PTT‐associated gene‐targeted therapy to treat UM and eyeball infection as shown in **Scheme** **1**. Many in vitro cell and bacterial experiments were conducted. In addition, a UM orthotopic model and eyeball infection model were built to verify the in vivo effectiveness of the prepared platform. This study provides the first successful application of intelligently released gene‐targeted drugs encapsulated in injectable PTT hydrogels for ocular tumor therapy and novel insights into tumor treatment involving vital organs.

## 2 Results and Discussion

### 2.1 Preparation and Characterization of the CP@Au Hydrogel

**Figure 1:**
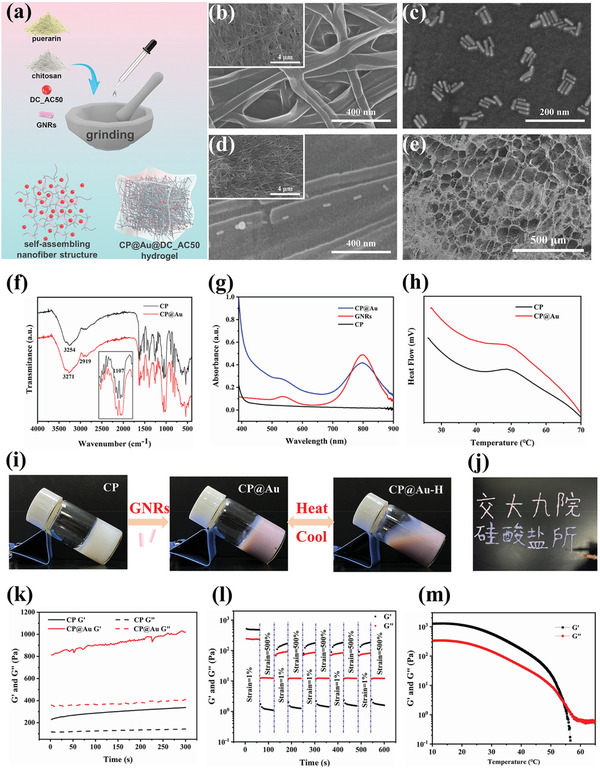
The preparation and characterization of the CP@Au hydrogel. a) Schematic diagram of the fabrication of the CP@Au hydrogel. SEM images of b) CP composite nanofiber hydrogel, c) gold nanorods, d) CP@Au hydrogel at high magnification, and e) CP@Au hydrogel at low magnification. f) FTIR spectra of CP and CP@Au hydrogels. g) UV–vis absorption spectrum of the CP hydrogel, GNRs solution, and CP@Au hydrogel. h) DSC curves of CP and CP@Au hydrogels. i) Photographs of the CP hydrogel, CP@Au hydrogel, and CP@Au sol at 50 °C. j) Photograph of the handwritten Chinese characters using the injectable CP@Au hydrogel. k) Time‐dependent rheological behavior of CP and CP@Au hydrogels at 1 Hz frequency and 1% strain. G′: storage modulus, G″: loss modulus. l) The shear‐thinning and self‐healing properties of the CP@Au hydrogel with recycled strain alternating between 1% and 500%. m) Temperature‐dependent gel‐sol transition of the CP@Au hydrogel.

During the process of eyeball injection treatment, a therapeutic hydrogel should be squeezed out from a specialized extremely fine needle and should remain at the UM position with a stable shape. Supramolecular self‐assembling hydrogels with a nanofiber structure and shear‐thinning ability are beneficial for loading drugs and injection. As illustrated in **Figure** **1**a, we developed a series of injectable and NIR smart controlled drug release CP@Au‐based hydrogels via a simple one‐step grinding method. The CP composite hydrogel and CP@Au hydrogel were formed rapidly after mixing with a minimal content of acetic acid aqueous solution under grinding. The formation of the CP@Au hydrogel was derived from chitosan@puerarin filaments assembled by hydrogen bonding, *π*–*π* stacking, hydrophobic interactions, van der Waals forces, and electrostatic interaction between positively charged CP hydrogel and GNRs (Figure S1, Supporting Information). These multiple noncovalent interactions, in turn, induced the growth of nanofibers and crosslinking of the network and were then devoted to the shear‐thinning and self‐healing ability of the CP@Au hydrogel. From scanning electron microscopy (SEM, Figure 1b–e) and transmission electron microscopy images (TEM, Figure S2, Supporting Information) we could clearly observe linear CP nanofibers with a diameter of ≈50–100 nm, columnar GNRs with a uniform size of ≈50 nm length and ≈10 nm width as well as the typical porous structure of CP@Au hydrogel with GNRs fixed in the nanofibers. The nanofibers gradually fractured with increasing amounts of GNRs (Figure S3, Supporting Information), which was probably due to the strong stacking between GNRs and nanofibers tearing nanofibers apart. Notably, the approximate concentrations of Au in CP@Au‐1, CP@Au‐2, and CP@Au‐3 were 0.15, 0.3, and 1.5 µm, respectively.

The successful incorporation of GNRs in the hydrogel was further confirmed by Fourier transform infrared (FTIR) spectra. The wide absorption bands at 3500–3000 cm^−1^ related to O—H stretching and the peak at ≈1100 cm^−1^ attributed to the C—O band of puerarin. From Figure 1f, we could see that with the incorporation of GNRs, there was a shift towards a higher wavenumber from 3254 to 3271 cm^−1^ and the peak at 1107 cm^−1^ disappeared. The difference between the FTIR spectra of CP and CP@Au hydrogel might suggest that the interactions between GNRs and CP nanofibers would influence the association degree of the CP composite. The X‐ray diffraction (XRD) patterns are shown in Figure S4, Supporting Information, all samples were semicrystalline composite, and the semicrystalline peaks changed after the mixing of GNRs, indicating that the hydrogel bearing GNRs possessed varies crystalline states. The typical UV–vis absorption peaks of prepared GNRs and CP@Au were detected at ≈800 and ≈520 nm (Figure 1g). Moreover, differential scanning calorimetry (DSC) analysis exhibited the closed denaturation temperature of the CP and CP@Au hydrogels, indicating that the hydrogel could transform into sol at 50 °C (Figure 1h). The resulting CP@Au hydrogel was an adorable milky pink, inheriting a similar gel‐sol transformation through a heating–cooling procedure but being distinguishing color from the CP hydrogel (Figure 1i). Interestingly, the CP@Au hydrogel could rapidly recover even when squeezed through a fine needle (Figure 1j and Movie S1, Supporting Information), which is advantageous for eyeball injection.

The incorporation of GNRs was expected to improve the stability and mechanical strength of the CP@Au hydrogel. To investigate the viscoelastic properties of hydrogels, the changes in the storage modulus (G′) and loss modulus (G″) of CP and CP@Au hydrogels were analyzed. G′ was greater than G″ when CP@Au was immediately prepared (Figure S5, Supporting Information), and G′ and G″ increased for a continuous 6 h test under 1% strain and 1 Hz frequency, indicating the formation of hydrogel and noncovalent interactions among nanofibers. The G′ of the CP@Au hydrogel increased from 300 to 1000 Pa and G″ increased from 100 to 200 Pa over 6 h. Moreover, the G′ and G″ of hydrogels were improved with increasing GNRs content (Figure 1k and Figure S6, Supporting Information) and CP@Au was more stable under the pressure similar to intraocular pressure (Figure S7, Supporting Information), illustrating that GNRs could reinforce the mechanical strength of the CP series of hydrogels, which was closely related to the fixed GNRs on the nanofiber. On the basis of network failure at a shear strain of 10% (Figure S8, Supporting Information), a recycled dynamic modulus assay was applied with a switched strain between 500% and 1%. From Figure 1l, G′ is less than G″ under 500% strain but greater than G″ under 1% strain, which reveals the breakage and recovery of the network structure.^[^ ^40^ ^]^ The results showed that the failed structure recovered rapidly and displayed normal hydrogel behavior over five cycles of breaking and reforming.

To determine the relationship between temperature and hydrogel rheological properties, the variations in G′ and G″ were tested from 10 to 70 °C (Figure 1m). Owing to the failure of the network structure originating from reversible noncovalent interactions, including hydrogen bonding and *π*–*π* stacking, G′ and G″ decreased gradually at a relatively low temperature and then dropped quickly. The point of intersection in the G′ and G″ temperature curves was ≈53 °C, approaching the denaturation temperature of DSC completion, which corresponds to the gel‐sol transformation point. To further study the thermosensitivity of CP@Au hydrogels, the changes in G′ and G″ were analyzed under a slow heating–cooling process with a variable temperature rate of 1 °C min^−1^. The results revealed that the G′ intersection points of CP and CP@Au were 27 and 37 °C (Figure S9, Supporting Information), and then the mechanical strength was substantially improved because of the reinforcement of GNRs and the orientation of hydrogen bonds. In summary, the mechanical strength of the CP@Au hydrogel can be conveniently adjusted through a heating–cooling procedure or by changing the content of GNRs. Consequently, the further confirmed mechanical stability, injectability, self‐healing, and thermosensitive ability of CP@Au hydrogels are promising for in vivo injection with the goal of drug delivery and controlled release.

### 2.2 Photothermal Effects of the CP@Au Hydrogel

**Figure 2:**
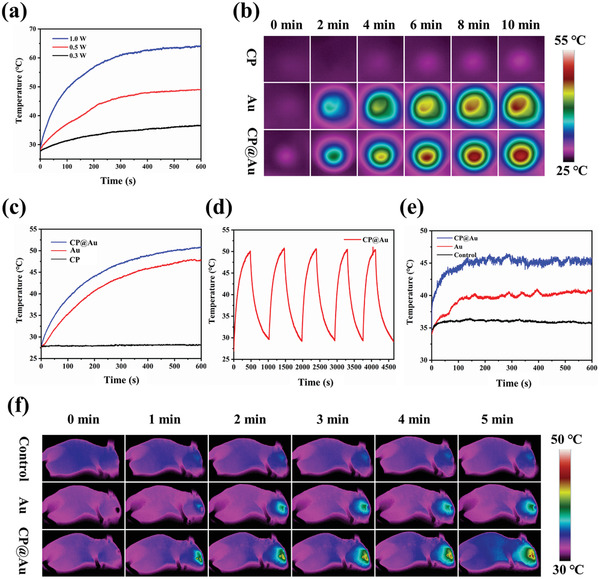
The photothermal effects of the CP@Au hydrogel. a) Temperature change of CP@Au hydrogel under 808 nm NIR light irradiation for 10 min with a power density of 0.3, 0.5, and 1.0 W cm^−2^. b) Infrared thermal images of the CP hydrogel, GNRs solution, and CP@Au hydrogel under 0.5 W cm^−2^ laser irradiation. c) Corresponding temperature change curves. d) Temperature variations of the CP@Au hydrogel for 5 on/off cycles. e) Temperature change curves of eyeballs injected by samples under 0.5 W cm^−2^ laser irradiation for 5 min. f) Corresponding infrared thermal images in vivo.

To investigate the photothermal effects of the CP@Au hydrogel, CP@Au hydrogels were exposed to 808 nm laser irradiation for 10 min. Temperature increase behaviors related to both laser density and GNRs content were clearly observed. It is widely recognized that eyes are fragile, high‐temperature (above 50 °C) PTT and large quantities of nanoparticles can probably injure eyeball tissues. Thus, a lower content of GNRs and lower power of NIR light density for lower‐temperature PTT treatment (42–47 °C) are needed.^[^ ^14^ ^]^ Under the power density of 0.3, 0.5, and 1.0 W cm^−2^ irradiation for 10 min, the temperatures of the CP@Au hydrogel increased from 27 to 35, 48, and 65 °C (**Figure** **2**a and Figure S10, Supporting Information). To avoid necrosis of normal tissues, CP@Au‐2 (referred to as CP@Au) and a laser density of 0.5 W cm^−2^ were selected for UM treatment in subsequent experiments on account of the lower GNRs concentration and higher photothermal conversion efficiency under NIR light irradiation. Because nanofiber hydrogels had better heat preservation characteristics than liquid solution, the temperature of CP@Au was slightly improved compared with the same concentration of GNRs in vitro (0.5 W cm^−2^, 10 min), and CP@Au possessed excellent cycled photothermal conversion properties (Figure 2b–d). However, there was a magnificent difference between the GNRs solution and CP@Au hydrogel in vivo tests. After 5 µL samples were injected into the eyeballs of mice, the temperature of eyeball alone (control) showed a slight upward trend and the GNRs groups improved from 33 to 40 °C, whereas the CP@Au groups showed an increase from 33 to 46 °C (0.5 W cm^−2^, 5 min, Figure 2e,f). All the results demonstrated that the CP@Au hydrogel could maintain outstanding photothermal conversion effects and was suitable for eyeball PTT treatment.

### 2.3 Controlled Drug Release Properties of the CP@Au Hydrogel and Gene‐Targeted Drug Selection

**Figure 3:**
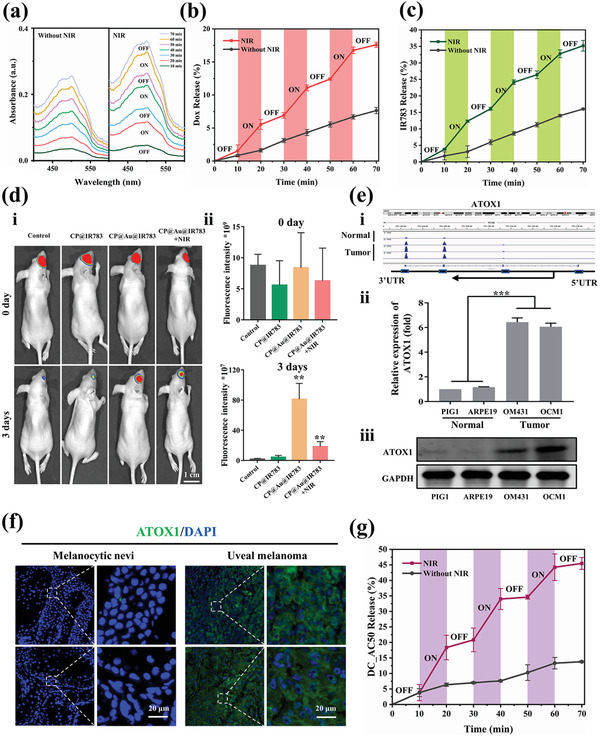
NIR light‐controlled drug release of the CP@Au series of hydrogels. a) UV–vis absorption spectrum of Dox release from CP@Au@Dox hydrogel without NIR light and with “ON‐OFF” irradiation. b) Corresponding percent of Dox release from the CP@Au@Dox hydrogel. c) NIR light triggered IR783 release behavior from CP@Au@IR783. d) IR783 release behavior in vivo. d‐i) Fluorescence images of mice injected with 5 µL of IR783 solution, CP@IR783, CP@Au@IR783 hydrogel, and CP@Au@IR783 under three consecutive days of irradiation (0.5 W cm^−2^, 5 min) at 0 and 3 days. d‐ii) Corresponding quantitative fluorescence intensity of samples. e) Aberrantly high expression of ATOX1 in tumor cells compared with paired normal cells. e‐i) Genome‐wide RNA sequencing of ATOX1. The black rectangles represent the exons of ATOX1. e‐ii) Real‐time PCR analysis of ATOX1 expression in UM cell lines (OCM1 and OM431) compared with normal cell lines (ARPE19 and PIG1). e‐iii) Western blot assay of ATOX1 in different cells. f) Immunohistochemical staining images of normal and tumor tissue specimens. Cell nuclei were blue, and ATOX1 was green. g) NIR light triggered DC_AC50 release behavior from the CP@Au@ DC_AC50 hydrogel. Data were expressed as the mean ± standard deviation (SD), *n* = 3, values were analyzed by were analyzed by unpaired *t* test, *p <0.05, **p <0.01, and ***p <0.001.

The admirable thermosensitive and photothermal effects of the CP@Au hydrogel inspired us to explore the release behavior of therapeutic drugs under NIR light irradiation. To determine whether the drug could be controllably released, a widely used drug, doxorubicin hydrochloride (Dox), was encapsulated in the CP@Au hydrogel, and the release behaviors of CP@Au@Dox before and after NIR irradiation (0.5 W cm^−2^) were investigated. Compared with less than 8% Dox release after 70 min incubation without irradiation, the cumulative release reached 20% in the CP@Au@Dox hydrogel, where a significant NIR‐triggered “on‐off” phenomenon appeared after irradiating three times (**Figure** **3**a,b). The NIR‐responsive Dox release of CP@Au@Dox was related to its NIR‐triggered gel‐sol transformation. Dox was tightly entrapped in the CP@Au hydrogel and difficult to diffuse out due to the multiple noncovalent interactions between Dox and nanofibers. The gel–sol transition occurred under irradiation, which led to the breakage of physical crosslinking, thereby accelerating drug diffusion from the loosened network. Importantly, the GNRs were prevented from leaking out of the hydrogels as a result of the interaction between GNRs and nanofibers (Figure S11, Supporting Information).

To further confirm the “on‐off” release behavior, the fluorescent agent IR783 was loaded in the CP@Au hydrogel and similar results were observed (Figure 3c). Animal experiments were carried out to study the controlled drug release properties of the CP@Au@IR783 hydrogel in vivo. The release tests were conducted as follows: mice were randomly divided into four groups, and the eyeballs of the mice were treated with 5 µL IR783 (aqueous solution), CP@IR783, CP@Au@IR783, or CP@Au@IR783+NIR. Drug distribution in vivo was monitored using a live imaging system by detecting IR783 fluorescence. There was no difference in fluorescence intensity between these groups immediately after sample injection (Figure 3d). Then, the CP@Au@IR783+NIR group was irradiated for three consecutive days (0.5 W cm^−2^, 5 min). The results demonstrated that IR783 encapsulated by the CP@Au hydrogel was unlikely to be cleared compared with the “free IR783” and “CP hydrogel encapsulated” condition, and NIR light irradiation could accelerate IR783 diffusion from the inside to the outside of the CP@Au@IR783 hydrogel. With the help of the CP@Au hydrogel, it is convenient to maintain the local drug concentration and duration of action; on the other hand, on‐demand drug release could be realized.

Traditional broad‐spectrum chemotherapeutic drugs such as Dox,^[^ ^41^ ^]^ paclitaxel,^[^ ^42^ ^]^ and cisplatin^[^ ^43^ ^]^ often induce damage in normal cells because of their poor specificity. As a visual organ, the eyeball is vulnerable and difficult to restore once injured. Moreover, studies have shown that UM is insensitive to traditional chemotherapy drugs.^[^ ^1^, ^5^ ^]^ To realize better therapeutic efficacy, drugs should meet the requirements of high sensitivity and specificity. Small‐molecule inhibitors could target aberrantly expressed genes and then kill tumor cells specifically without harming normal tissues through gene‐targeted therapy, which has recently received extensive attention.^[^ ^37^, ^38^ ^]^ To identify aberrant genes expressed in tumors, we compared gene expression profiles between UM cells and paired normal cells that we previously reported, which can be accessed at the Gene Expression Omnibus database under accession number GSE137675.^[^ ^44^ ^]^ Genome‐wide RNA sequencing analysis showed that ATOX1 was highly expressed in UM cells compared with paired normal cells (Figure 3e‐i). ATOX1 encodes a copper chaperone and plays an important role in tumorigenesis.^[^ ^39^, ^45^ ^]^ Kaplan–Meier survival analysis of melanoma patients in Gene Expression Profiling Interactive Analysis (GEPIA) confirmed that ATOX1 is negatively correlated with patient prognosis (Figure S12, Supporting Information). Therefore, it is reasonable to believe that ATOX1 is a potential target for gene‐targeted therapy of UM. To further confirm the aberrant expression of ATOX1 in UM, we performed polymerase chain reaction analysis (PCR) and Western blot to verify the conclusions above. The real‐time quantitative PCR and Western blot results revealed that the relative expression of ATOX1 in tumor cells (OM431 and OCM1) was significantly higher than that in normal cells (PIG1 and ARPE19), which was consistent with genome‐wide RNA sequencing results (Figure 3e‐ii,iii). In addition, we collected tumor tissue specimens from UM patients and then carried out immunohistochemical staining. Figure 3f indicates abnormally high expression of ATOX1 in UM tissue compared with paired normal tissues. Based on these results, we confirmed the aberrantly high expressions of ATOX1 at RNA, protein, and tissue levels. Therefore, the key for gene‐targeted therapy is to find a specific drug that can inhibit the expression of ATOX1. According to previous research, DC_AC50, as a small‐molecule inhibitor, could specifically target ATOX1 and induce the death of tumor cells, and was selected to be encapsulated in the CP@Au hydrogel for UM treatment.^[^ ^39^ ^]^ The properties of CP@Au hydrogel were not affected obviously with DC_AC50 loading (Figure S13, Supporting Information). The release activities of DC_AC50 mixed into the CP@Au hydrogel displayed sustained and NIR light‐responsive controlled release behavior over three irradiation cycles, which demonstrated the successful loading of DC_AC50 in the CP@Au hydrogel and the universal controlled release property of the CP@Au hydrogel (Figure 3g).

### 2.4 Biocompatibility of the CP@Au Hydrogel

**Figure 4:**
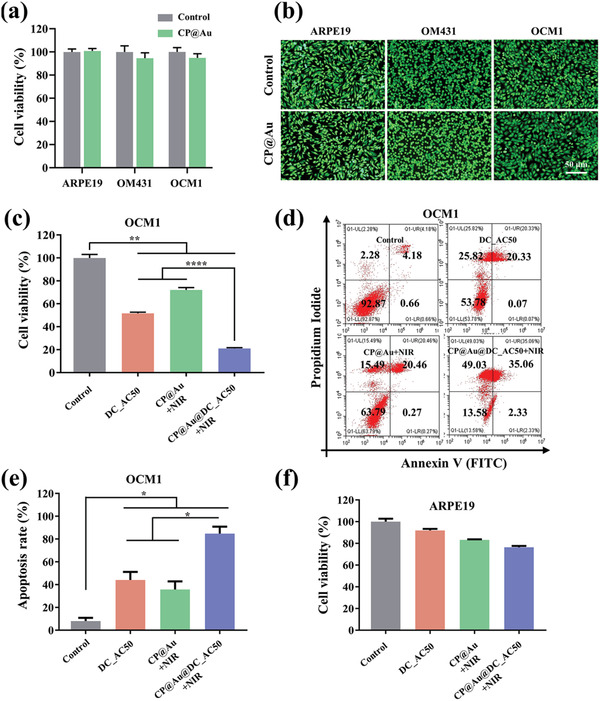
Biocompatibility evaluation and antitumor effect in vitro. a) CCK8 assay of normal cells (ARPE19) and tumor cells (OM431, OCM1) cocultured without material (control) and with CP@Au hydrogel. b) Live/dead staining of the cells treated with samples and the green fluorescence indicates live cells. c) CCK8 assay of OCM1 cells cocultured with DC_AC50, CP@Au+NIR, and CP@Au@DC_AC50+NIR for 24 h. Cells subjected to no treatment were set as the control group. d) Apoptosis analyses of OCM1 cells treated with samples for 24 h. e) Statistical histogram of the apoptosis assay. f) CCK8 assay of ARPE19 cells cocultured with samples for 24 h. Data were expressed as the mean ± standard deviation (SD), *n* = 3, values were analyzed by were analyzed by unpaired *t* test, *p <0.05, **p <0.01, and ****p <0.0001.

For any biomedical application, the primary concern is to investigate the biocompatibility of biomaterials. To investigate the biocompatibility of the CP@Au hydrogel, we carried out cell counting kit‐8 (CCK‐8) tests. Varied concentrations of GNRs and different amounts of CP@Au hydrogel showed excellent biocompatibility (Figure S14, Supporting Information). As presented in **Figure** **4**a and Figure S15, Supporting Information, the CCK‐8 assay showed that the CP@Au hydrogel did not inhibit the viabilities of normal cells (ARPE19) and tumor cells (OCM1, OM431) over 24 and 48 h, which was similar to the results of the control group without materials. Moreover, a live/dead staining test was conducted, and almost all of the cells cocultured with the CP@Au hydrogel remained alive (Figure 4b). Additionally, the hemolysis of the CP@Au group was measured, and the hemolysis ratios were negligible (Figure S16, Supporting Information). Taken together, these results confirmed the great biocompatibility of the CP@Au hydrogel.

### 2.5 Synergistic Antitumor Effect In Vitro

According to the photothermal conversion property and the smart release behavior of the gene‐targeted drug, the CP@Au@DC_AC50 hydrogel could be used for synergistic PTT and responsive gene‐targeted therapy against UM under NIR light. To verify the antitumor effect of the hydrogels under NIR and compare the efficacies of monotherapy with combination therapy, OCM1 was cocultured with DC_AC50 (gene‐targeted therapy), CP@Au+NIR (PTT), and CP@Au@DC_AC50+NIR (gene‐targeted therapy/PTT) for 24 h. Considering the cell sensitivity and safety of DC_AC50, the concentration of DC_AC50 solution and DC_AC50 encapsulated in hydrogel was set to 10 µm (Figure S17, Supporting Information), and two groups of hydrogels were irradiated with 0.5 W cm^−2^ NIR light for 5 min. The in vitro antitumor effect was measured by CCK‐8 assay, and the results showed that the viability of cells treated with DC_AC50 and CP@Au+NIR was 51.6% and 72.1%, respectively. Correspondingly, only 21% of cells were viable in the CP@Au@DC_AC50+NIR group (Figure 4c). In addition, we conducted a cell apoptosis assay (Figure 4d) to evaluate the synergistic antitumor effect of the treatments, and the statistical analysis results of the triple apoptosis assays are shown in Figure 4e, revealing that the death rate of OCM1 was the highest in the CP@Au@DC_AC50+NIR group. Furthermore, OM431, another kind of UM cell, was also applied and presented similar results, indicating that CP@Au@DC_AC50+NIR possessed excellent synergistic antitumor effects (Figures S18 and S19, Supporting Information). To confirm the safety of gene‐targeted therapy, normal ARPE19 cells in the eyeball were used to evaluate cell viability after different treatments. As shown in Figure 4f, the gene‐targeted drug DC_AC50 caused minimal damage to normal cells compared with tumor cells in the eyeball, and the combination therapy had little effect on ARPE19. These tests demonstrated that the synergistic antitumor efficiency of CP@Au@DC_AC50+NIR could not only efficiently inhibit the viability of UM cells but also possess reliable safety for normal cells, which was suitable for UM treatments.

### 2.6 Construction of the UM Orthotopic Model and Antitumor Effects In Vivo

**Figure 5:**
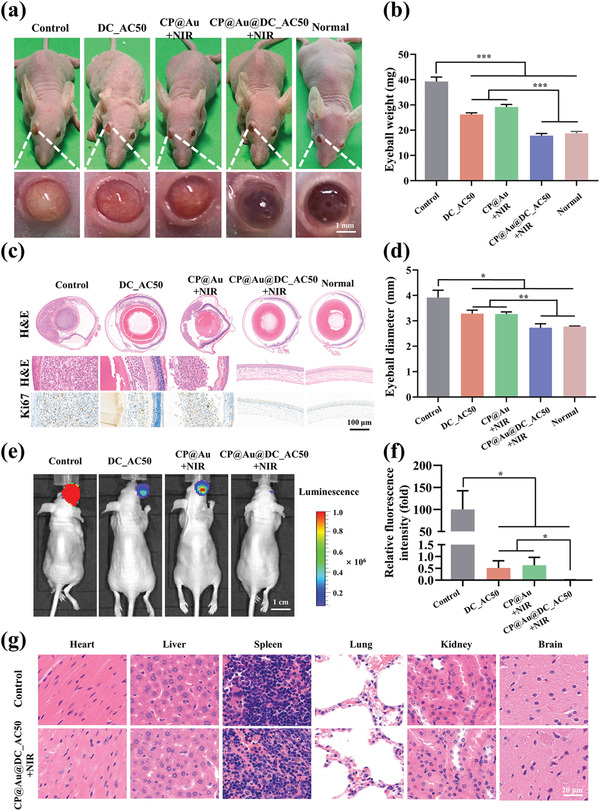
Antitumor effects of different treatments in vivo. a) Overall and eyeball appearance photos of the mice 2 weeks post treatments. b) Eyeball weight in different treatment groups. c) H&E and Ki67 staining images of eyeballs in different groups. d) Diameter of eyeball in these groups. e) Animal fluorescence imaging of mice 2 weeks post treatment. f) The corresponding statistical histogram of fluorescence intensity. g) H&E‐stained tissues including heart, liver, spleen, lung, kidney, and brain. Data were expressed as the mean ±SD, *n* = 6, values were analyzed by were analyzed by unpaired *t* test, *p <0.05, **p <0.01, and ***p <0.001.

To further investigate the synergistic antitumor effect of CP@Au@DC_AC50+NIR in vivo, we constructed an orthotopic model of UM to mimic the intraocular microenvironment in which UM grows. Briefly, OCM1 was injected into the eyeballs of mice. Five days later, four groups of mice were treated with 5 µL samples, including sterile phosphate buffer saline (PBS, control), DC_AC50, CP@Au+NIR, and CP@Au@DC_AC50+NIR, while the normal group accepted none of the treatments. Owing to the small volume of the eyeball, we reduced the total content of DC_AC50, and the concentration was set to 100 µm. The hydrogel groups were irradiated by 0.5 W cm^−2^ NIR light for three consecutive days for 5 min each time. **Figure** **5**a shows the external appearance of the eyeballs 2 weeks post treatment. Eyeballs in the control group treated with sterile phosphate buffer saline (PBS) were filled with tumor tissue, and obvious vascular proliferation as well as eyeball mobility disturbance could also be observed. Compared with the control group, the degree of tumor proliferation was relatively lower but the eyeball still appeared muddy and swollen in the DC_AC50 and CP@Au+NIR groups, indicating certain therapeutic benefits of gene‐targeted therapy and PTT alone. Remarkably, the eyeball of the CP@Au@DC_AC50+NIR group treated with synergistic gene‐targeted therapy/PTT was clear and without tumor proliferation, which was similar to the normal group.

To better visualize the proliferation of UM cells, eyeballs were subjected to hematoxylin and eosin (H&E) and Ki67 staining. Figure 5c demonstrates that tumor cells broke into the vitreous and massively proliferated inside the eyeball in the control group, and tumors proliferated outside the vitreous merely in the DC_AC50 and CP@Au+NIR groups. In contrast, in the CP@Au@DC_AC50+NIR group, the eyeball was pure and no tumor cells were observed inside. Eyeballs were collected, and we measured the weight (Figure 5b) and diameter (Figure 5d). The results revealed that the eyeballs of the CP@Au@DC_AC50+NIR group possessed the normal weight and diameter, verifying the excellent antitumor efficiency of gene‐targeted therapy/PTT. To quantitatively evaluate the therapeutic effect, OCM1 cells were labeled with luciferase and then used to construct an orthotopic mouse model of UM. Five days post OCM1/Luc cell injection, live imaging showed that there was no difference concerning the fluorescent signal between different groups (Figure S20, Supporting Information). Afterwards, mice were treated as previously described. Two weeks later, animal fluorescence imaging devices were used to judge the quantity of UM cells that received different treatments (Figure 5e,f). Compared with the control group, mice treated with DC_AC50 and CP@Au+NIR exhibited lower fluorescence intensity, and the relative values of the two groups were close to 1%. Moreover, the fluorescence of the CP@Au@DC_AC50+NIR group was hardly distinguishable. These conclusions were consistent with previous experiments. Although the biocompatibility of the CP@Au hydrogel had been tested in vitro previously, further toxicity tests in vivo are still needed. HE staining assays of major organs, including the heart, liver, spleen, lung, kidney, and brain, indicated that there was no toxicity in the CP@Au@DC_AC50+NIR group (Figure 5g). In addition, there was no difference in the body weight of the mice between different groups (Figure S21, Supporting Information). Above all, these results confirmed the excellent synergistic antitumor effects of gene‐targeted therapy/PTT with benefit of the CP@Au@DC_AC50 hydrogel under NIR light irradiation, emerging a novel method for UM treatment without any toxicity.

### 2.7 Antibacterial Activities In Vitro and In Vivo

**Figure 6:**
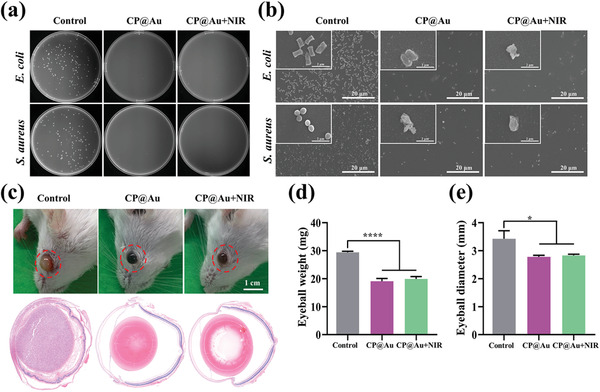
Antibacterial properties of the CP@Au hydrogel. a) Photographs of bacterial colonies of recultivated *E. coli* and *S. aureus* without treatment (control) and treated with the CP@Au hydrogel, CP@Au+NIR. b) SEM images of *E. coli* and *S. aureus* subjected to different treatments. c) Photos of *S. aureus*‐infected mice eyeballs without treatment (control) and with different treatments on the 5th day and of H&E‐stained eyeballs. The corresponding statistical diagrams of d) eyeball weight and e) eyeball diameter. Data were expressed as the mean ±SD, *n* = 3, values were analyzed by were analyzed by unpaired *t* test, *p <0.05 and ****p <0.0001.

Bacterial infection originating from invasive operation and the internal tumor could possibly lead to severe outcomes. To avoid the occurrence of infection, gram‐negative bacteria *Escherichia coli* and gram‐positive bacteria *Staphylococcus aureus* were applied to investigate the antibacterial ability of the injected materials. **Figure** **6**a presents typical photos of recultivated bacterial colonies treated with different samples. No colonies of the two kinds of bacteria could be observed after mixing the minimum CP@Au hydrogel with or without irradiation, but many colonies were detected in the control group. The amounts and statements of bacteria are shown in SEM images (Figure 6b). A large number of bacteria could be found, and the membrane structures of the two types of bacteria were integral in the control group. However, few bacteria could be found in the group treated with the CP@Au hydrogel, and the bacteria appeared abnormal. After irradiation, fewer bacteria were present, and their membranes were completely broken, indicating the death of bacteria. To further confirm the antibacterial effects of the resulting samples, an eyeball infection model in mice was constructed for in vivo tests. Five milliliters of *S. aureus* suspension (threefold dilutions of 10^8^ cfu/mL) was injected into the eyeballs, followed by 5 µL samples of each group. Five days post treatment, obvious bacterial infection was observed in the control group, and the eyeball became swollen. In contrast, the eyeballs of mice subjected to hydrogels were clean and clear (Figure 6c). HE staining tests further confirmed that large quantities of neutrophils fulfilled the eyeball of the control group, while no inflammation was found in the groups of the two hydrogels. The weight and diameter of the eyeballs are presented in Figures 6d and 6e, respectively. The proliferation of bacteria and inflammation were reflected by abnormally increased weight and diameter. Therefore, the outstanding antibacterial property of the CP@Au hydrogel could prevent infection during the process of antitumor treatment.

## 3 Conclusion

In this study, a self‐assembling nanofiber hydrogel containing a gene‐targeted drug was first constructed via a one‐step grinding method to treat UM and eyeball infection. Thanks to the reinforcement of GNRs incorporation, the hydrogel exhibited suitably improved mechanical strength for eyeball injection. Meanwhile, the NIR light triggered photothermal transition and gel‐sol transformation could realize mild PTT effect and smart drug release. NIR light‐triggered photothermal/gene‐targeted therapy of CP@Au@DC_AC50 exhibited brilliant synergistic antitumor efficiency in vitro and in vivo. Importantly, the controlled release of DC_AC50 and mild hyperthermia exhibited little influence on normal cells, which guaranteed the safety of intraocular tissue. Moreover, the excellent internal antibacterial ability of the hydrogel could prevent eyeball infection by invasive injection and bacteria harbored in the tumor. The constructed CP@Au@DC_AC50 thus emerges as a promising strategy for UM therapy by a “single injection, multiple treatment” method, and the gene‐targeted therapy/PTT/antibacterial platform has potential for future application in combating cancer.

## Conflict of Interest

The authors declare no conflict of interest.

## Supporting information

Supporting Information

Supplemental Movie 1

## Data Availability

The data that support the findings of this study are openly available in Gene Expression Omnibus database at http://doi.org/10.1186/s12943-019-1088-x, GSE137675.
