# Supplementary material for: A Novel Stimuli‐Responsive Injectable Antibacterial Hydrogel to Achieve Synergetic Photothermal/Gene‐Targeted Therapy towards Uveal Melanoma
Source: Adv Sci (Weinh). 2021 Jul 31;8(18):2004721. doi: 10.1002/advs.202004721 (PMC8456278; doi:10.1002/advs.202004721)
Supplement: Supplementary file 1 — Supporting Information [file ADVS-8-2004721-s001.pdf]

© 2021 Wiley-VCH GmbH

ADVANCED SCIENCE Open Access

Supporting Information

for *Adv. Sci.,* DOI: 10.1002/advs.202004721

A Novel Stimuli-responsive Injectable Antibacterial Hydrogel to Achieve Synergetic Photothermal/Gene-targeted Therapy towards Uveal Melanoma

Shaoyun Wang, Baohui Chen, Liping Ouyang, Donghui Wang, Ji Tan, Yuqin Qiao, Shengfang Ge, Jing Ruan, Ai Zhuang, Xuanyong Liu*, and Renbing Jia**

Supporting Information

A Novel Stimuli-responsive Injectable Antibacterial Hydrogel to Achieve Synergetic Photothermal/Gene-targeted Therapy towards Uveal Melanoma

Shaoyun Wang, Baohui Chen, Liping Ouyang, Donghui Wang, Ji Tan, Yuqin Qiao, Shengfang Ge, Jing Ruan, Ai Zhuang, Xuanyong Liu*, and Renbing Jia**

Experimental Section

Synthesis of gold nanorods (GNRs)

For this purpose, 0.12 mL of 0.02 M chloroauric acid ($HAuCl_4$, Aladdin, China) solution, 5 mL of 0.1 M cetyltrimethylammonium bromide solution (CTAB, Aladdin,

China) and 2.5 mL of distilled water were mixed with the help of magnetic stirring. Then, 0.6 mL of 0.01 M sodium borohydride ($NaBH_4$, Aladdin, China) solution near its freezing point was added, and the solution turned from deep yellow to brown. Stirring was continued for 2 min to obtain the GNR seed solution. The prepared solution was obtained after standing for 2-5 h at a temperature of 27-30°C. Next, 15 mL of 0.1 M CTAB, 0.375 mL of 0.01 M $AgNO_3$ (Aladdin, China) and 0.75 mL of 0.02 M $HAuCl_4$ were mixed, and the mixture quickly turned red under magnetic stirring. Then, 0.1 M ascorbic acid (Aladdin, China) solution was added dropwise until the solution began to fade. The solution was stirred for 2 min to obtain the growth solution of GNRs, and 72 μL of seed solution was added to the growth solution. Stirring was continued for another 2 min. The solution was left to stand for 12-24 h at 25-27°C to obtain the GNRs.

Synthesis of the injectable CP hydrogel and CP@Au hydrogel

The CP hydrogel was directly prepared via a simple grinding method. Briefly, 50 mg of sterilized chitosan (deacetylation degree 95%, 400 mPa.s, Aladdin, China) and 100 mg of puerarin (Aladdin, China) were ground by mortar and pestle in the presence of 300 μL of 0.5 wt% acetic acid (Sinopharm Group, China) for 5-10 min until the acetic acid was completely volatilized. Nine milliliters of ultrapure water was added to disperse the composite of chitosan@puerarin, and grinding was continued until a white gel was obtained. One milliliter of 0.5% acetic acid was added, and a viscous and shaped CP hydrogel was formed immediately. The mixture was allowed to stand for 12 h at room temperature to obtain the composite of CTS, PUE and Au nanorods referred to as CP@Au.

GNRs loading: One milliliter of GNRs was collected and centrifuged twice at 10,000 rpm at room temperature for 20 min and then redispersed with 100 μL of ultrapure water. Next, 0.5 mL of GNRs aqueous solution was mixed into the chitosan@puerarin composite under grinding (CP@Au). The CP@Au hydrogel was synthesized as previously described. The CP hydrogels loaded with 0.25 mL, 0.5 mL

and 2.5 mL of GNRs solution were denoted as CP@Au-1, CP@Au-2 and CP@Au-3, respectively.

Sample characterizations of the CP@Au hydrogel

Field emission scanning electron microscopy (FE-SEM; Magellan-400, Hitachi, Japan) and transmission electron microscopy (TEM, S-3400N Type I, Hitachi, Japan) were used to analyze the morphology and structure of the samples. The samples for TEM observation were freeze-dried, diluted with ultrapure water, and then dropped onto a copper foil net and air-dried. Fourier transform infrared spectroscopy (FTIR; Tensor 27, Bruker, Germany) was recorded at wavelengths ranging from 4000 to 400 cm^{-1}. Differential scanning calorimetry (DSC; Netzsch STA 409 PC Luxx, Selb, Germany) measurements were carried out on hydrogels containing different amounts of GNRs. The temperature of the samples was raised from 25 to 70°C at a rate of 10°C min^{-1} under a N_2 atmosphere. The construction of the GNRs and GNRs in CP hydrogel was characterized by ultraviolet and visible spectroscopy (UV-vis; Lambada 750, PerkinElmer, USA).

Rheological test

The rheological properties of the hydrogel were assessed on a rheometer (MCR 301, Anton Parr, Austria) at 25°C. One milliliter of prepared hydrogel samples was injected on the center of a 25 mm diameter parallel plate with a suitable gap. Modulus tests were applied at a constant strain rate of 1% and frequency of 1 Hz. Strain-sweep measurements were performed over a range from 1% to 1000% strain. The self-healing performance was analyzed by dynamic modulus assay with the strain switched between 500% and 1% for five times. Dynamic temperature sweeps were carried out ranging from 10 to 70°C at a rate of 1°C min^{-1}.

Photothermal effect measurements *in vitro* and *in vivo*

An infrared thermal imager (Fotric 285s, USA) was used to record the temperature changes in the CP hydrogel, GNRs aqueous solution and CP@Au

hydrogel *in vitro*. Samples (0.5 mL) were placed in a 24-well plate and then irradiated with an 808 nm near-infrared (NIR) laser at a density of 0.5 W/cm^2 for 10 min. The temperature changes in different amounts of GNRs in CP@Au hydrogels exposed to 0.3 and 1.0 W/cm^2 NIR irradiation were also measured.

To investigate the photothermal effect of the CP@Au hydrogel *in vivo*, 5 μL of GNRs aqueous solution and CP@Au hydrogel were injected into the eyeballs of BALB/c nude mice (female, 5 weeks old) through the sclera using a Hamilton syringe (Hamilton company, Reno, NV, USA). Then, the temperature change in the eyeballs under 0.5 W/cm^2 NIR irradiation for 5 min was recorded.

Drug loading and release by NIR smart controlled release system

One milliliter of 1000 μM doxorubicin hydrochloride (Dox, Aladdin, China) was mixed into CP composite-like GNRs, and 8 mL of deionized water and 1 mL of 0.5% acetic acid were subsequently added. The CP@Au@Dox hydrogel was prepared under continuous grinding with a concentration of 100 μM Dox. The release of Dox from the CP@Au@Dox hydrogel was determined in DI water. One milliliter of DI water was placed in a 24-well plate above 1 mL of hydrogel. The hydrogel was exposed to an 808 nm NIR laser (0.5 W/cm^2) for 10 min followed by an interval of 10 min, and the procedure was repeated three times. Afterwards, the amount of Dox released from the collected solutions was determined by UV-vis spectroscopy. In comparison, the hydrogel without NIR light irradiation was set as a control. In addition, the fluorescent reagent IR783 (Aladdin, China) or the gene-targeted drug DC_AC50 (APExBIO, USA) was encapsulated in the CP@Au hydrogel as previously described with the same concentration of 100 μM, and the NIR smart controlled release behavior was assayed as well.

To confirm the controlled release ability of the CP@Au hydrogel *in vivo*, 5 μL doses of 100 μM IR783 aqueous solution, CP@Au@IR783 and CP@Au@IR783+NIR samples were injected into the eyeballs of BALB/c nude mice (female, 5 weeks old). Bioluminescence intensity was measured with a Spectral

Instruments Imaging Optical Imaging Platform (Lago X, Cold Spring Biotech Corp.; 20 s exposure) after three consecutive days of irradiation under 808 nm NIR light (0.5 W/cm^2, 5 min). The average signal intensities within a circular region of interest (ROI) were quantified.

Cell culture

The human UM cell lines OCM1 and OM431 were cultured in DMEM (GIBCO, Carlsbad, CA, United States) supplemented with 10% certified heat-inactivated fetal bovine serum (FBS; GIBCO), penicillin (100 U/mL), and streptomycin (100 mg/mL) at 37°C in a humidified 5% CO_2 atmosphere. Adult retinal pigment epithelial cell line-19 (ARPE19) and human melanocyte cell line PIG1 were cultured in RPMI 1640 medium (GIBCO, Carlsbad, CA, United States) supplemented with 10% certified heat-inactivated fetal bovine serum (FBS; GIBCO), penicillin (100 U/mL), and streptomycin (100 mg/mL) at 37°C in a humidified 5% CO_2 atmosphere.

Uveal melanoma tissue microarray and immunohistochemistry

UM tissues were obtained from the Ninth People's Hospital of Shanghai JiaoTong University School of Medicine. This experiment was permitted by Ethical Committee of the Ninth People's Hospital of Shanghai JiaoTong University School of Medicine. The informed consent of all patients was obtained. The immunohistochemistry staining for the UM tissue microarray (TMA) slide was performed by the following steps. The slide was deparaffinized, rehydrated, immersed in citrate-buffered saline and boiled for 20 min, and then blocked with 10% goat serum. The slide was incubated with anti-ATOX1 antibody (Abcam, UK) and then with secondary antibody (Abcam, UK).

Western blot

Cells were harvested and rinsed with PBS. Cell extracts were prepared with lysis buffer and centrifuged for 20 min at 10,000 ×g and 4 °C. Protein samples were

separated by 7.5% (wt/vol) sodium dodecyl sulfate–polyacrylamide gel electrophoresis (SDS-PAGE) and transferred to polyvinylidene fluoride membranes. After blocking with 5% milk for 2 h at room temperature, the membrane was incubated with 3 μg/mL of antibody in 5% BSA overnight at 4°C. The membrane was then incubated with secondary antibody. The band signals were visualized and quantified by an Odyssey Infrared Imaging System (LI-COR, USA).

Quantitative real-time PCR

RNA was extracted with an EZ-press RNA Purification Kit (B0004), and cDNA was generated by a PrimeScript RT Reagent Kit (Takara). Quantitative real-time PCR was performed to identify ATOX1 expression. The housekeeping gene GAPDH was used as an endogenous control.

Biocompatibility of CP@Au hydrogel

The biocompatibility of the CP@Au hydrogel was assessed by CCK8 and live/dead staining assays. To investigate the proliferation rate of normal cells (ARPE19) and tumor cells (OM431 and OCM1) incubated with CP@Au hydrogel, 3×10^4 cells were seeded in 24-well plates containing 1 mL of medium. Twenty-four hours later, a transwell loaded with 1 mL of CP@Au hydrogel was placed in the plate for 24 h and 48 h separately at 37°C under a 5% carbon dioxide atmosphere. Then, 100 μL of CCK8 solution (Dojidon, Kumamoto, Japan) was added to the plates for 4 h. A microplate reader (ELX800, BioTec, Winooski, VT, United States) was employed to measure the absorbance of samples at 450 nm. A live/dead staining assay was carried out to evaluate the viability of the cells. Cells treated with CP@Au for 24 h were stained with a LIVE/DEAD staining kit and analyzed under a confocal laser scanning microscope (CLSM; Olympus, Japan).

Antitumor effects *in vitro*

To evaluate the antitumor ability of all samples (Control, DC_AC50, CP@Au+NIR and CP@Au@ DC_AC50+NIR) *in vitro*, a CCK8 assay and flow cytometry were applied. The concentration of DC_AC50 solution and DC_AC50 encapsulated in hydrogel was 10 μM. A total of 3×10^4 tumor cells, including OM431 and OCM1, were seeded in 24-well plates overnight separately. All samples were transferred into plates, and two groups of hydrogels were irradiated by 0.5 W/cm^2 NIR for 5 min. The materials and cells were then cocultured for another 24 h. Cells were treated with CCK8 kits, and the results were measured. The activity of normal cells incubated with all samples was also tested.

In addition, cells (3×10^4) were seeded in 24-well plates and treated by the same methods previously described. Then, a cell suspension was made by digestion with 0.25% trypsin without EDTA, followed by washing with PBS. Apoptotic cell death was identified by double staining with Annexin V-fluorescein isothiocyanate (FITC) and propidium iodide (BD Biosciences, USA). The double-stained apoptotic cells were measured by a flow cytometer (FACS Calibur, BD Biosciences, USA) using FlowJo software for acquisition and analysis.

Antitumor activity *in vivo*

Orthotopic tumor models were built to evaluate the antitumor ability of these samples (Control, DC_AC50, CP@Au+NIR and CP@Au@ DC_AC50+NIR) *in vivo*. Animal experiments were conducted in accordance with the animal policies of Shanghai JiaoTong University. BALB/c nude mice (female, 5 weeks old) were used for orthotopic ocular tumor construction. Five microliters of medium containing 2×10^5 OCM1 cells was injected into the vitreous of eyes. Five days later, mice were treated with 5 μL of sterile PBS (control), DC_AC50, CP@Au+NIR or CP@Au@DC_AC50+NIR. The concentration of DC_AC50 was 100 μM, and the hydrogel groups were irradiated with 0.5 W/cm^2 NIR for three consecutive days for 5 min each time. Two weeks after treatments, all mice were photographed and sacrificed, and the eyeballs were collected. A caliper and electronic balance were used to measure the diameter and weight of the eyeballs. Then, harvested eyeballs were fixed with 10%

neutral buffered formalin, embedded in paraffin, and cut into 4 μm thick sections. The tissue sections were immunostained using antibodies against hematoxylin and eosin (H&E) and Ki-67 according to the manufacturer's instructions (Bioworld Technology, Nanjing, China) and observed under an optical microscope (Leica TE2000-S microscope, Tokyo, Japan). Organs including the heart, liver, spleen, lung, kidney and brain were stained with H&E.

Bioluminescence imaging for evaluation of antitumor efficacy *in vivo*

OCM1/Luc cells were injected into the eyeballs of the mice, and the subsequent manipulation was similar to that described above. Two weeks after treatment, 150 mg/kg firefly luciferin (Thermo Fisher Scientific, China) was injected into the mice. Fifteen minutes later, the mice were anesthetized with 200 μL of 1% pentobarbital sodium solution. Bioluminescence was measured with a Spectral Instruments Imaging Optical Imaging Platform (Lago X, Cold Spring Biotech Corp.; 20 s exposure). The average signal intensities within a circular ROI were quantified.

Antibacterial activity *in vitro* and *in vivo*

Bacterial counting and SEM observation using gram-positive *Staphylococcus aureus* (*S. aureus*, ATCC 25923) and gram-negative *Escherichia coli* (*E. coli*, ATCC 25922) were applied to evaluate the antibacterial ability of the CP@Au hydrogel. Then, 20 μL of hydrogel was added to glass slides in a 24-well plate followed by 0.5 mL of 10^7 cfu/ml bacterial suspension. Twenty-four hours later, the different groups of bacteria were separated from the slides and inoculated into a standard agar culture medium for bacterial counting. After culturing for 18 h in the incubator, images of the bacterial colonies were taken by a gel imaging system (Protein Simple, USA). To observe the bacterial topography, 2.5% glutaraldehyde solution was utilized to fix the bacteria on the slides overnight, and then the slides were dehydrated with gradient ethanol solutions and hexamethyl disilazane ethanol solutions.

BALB/c mice (female, 5 weeks old) were used for the evaluation of anti-infection ability *in vivo*. Five-microliter suspensions of sterile PBS containing $1/3\times10^8$ cfu/mL *S.*

aureus were injected into the vitreous of eyeballs. Each mouse was subjected to 5 μL of sterile PBS (control), CP@Au or CP@Au+NIR treatment immediately. Five days post treatment, all mice were photographed and sacrificed, and the eyeballs were collected. These eyeballs were treated the same way.

Data analysis

All statistical analyses were conducted with a GraphPad Prism 5 statistical software package. All of the data were expressed as the mean ± standard deviation (SD). Statistically significant differences (P) were analyzed by unpaired t test. A value of $p<0.05$ was considered statistically significant and was represented by the symbol "*"; a value of $p<0.01$ was represented by "**"; and $p<0.001$ was "***".

Supplemental figures

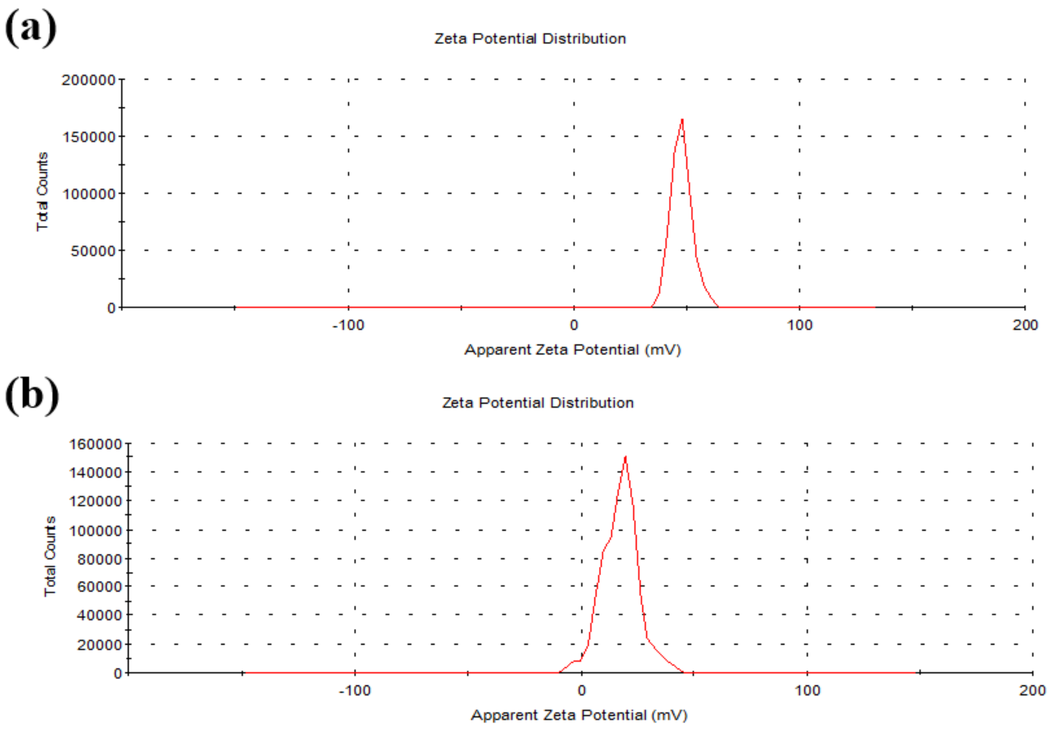

Figure S1. Zeta potential of (a) CP hydrogel and (b) GNRs. Both CP hydrogel and GNRs were all positively charged.

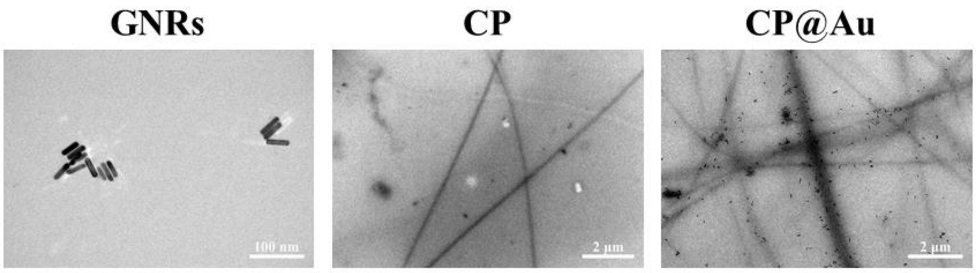

Figure S2. TEM images of GNRs, CP hydrogel and CP@Au hydrogels.

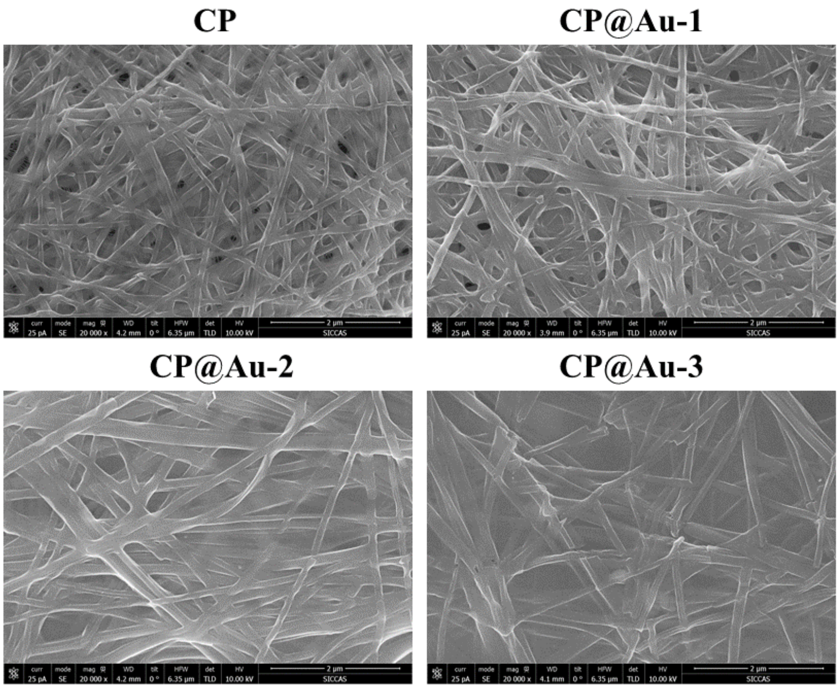

Figure S3. SEM images of the CP and CP@Au series of hydrogels. The concentration of Au element in CP@Au-1, CP@Au-2 and CP@Au-3 was 0.15 μM, 0.3 μM and 1.5 μM, respectively. The nanofibers gradually fractured with increasing amounts of GNRs.

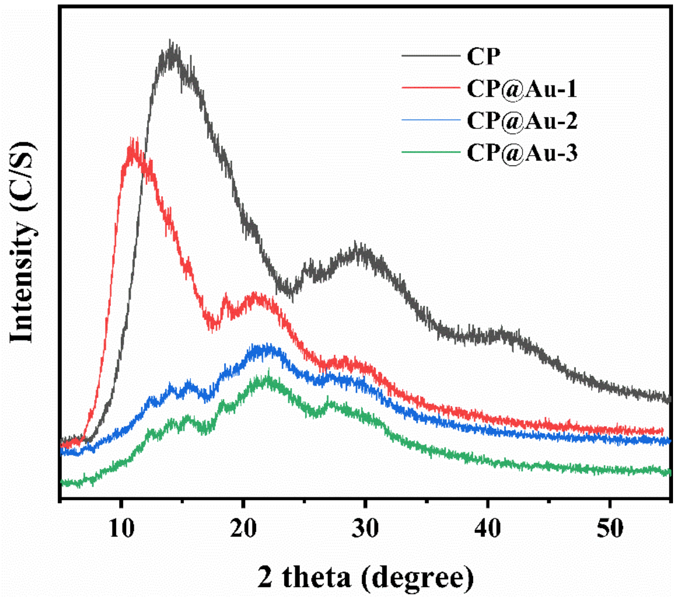

Figure S4. XRD patterns of CP and CP@Au hydrogels.

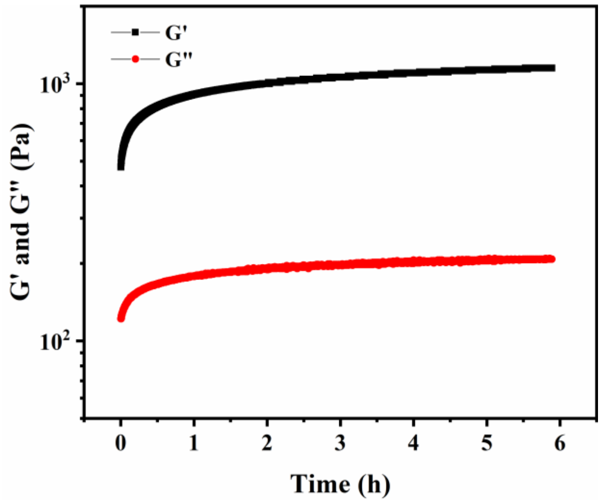

Figure S5. Rheological properties of the CP@Au hydrogel for continuous 6 h test under 1% strain and 1 Hz frequency, and the G’ represent the storage modulus and G” represent the loss modulus.

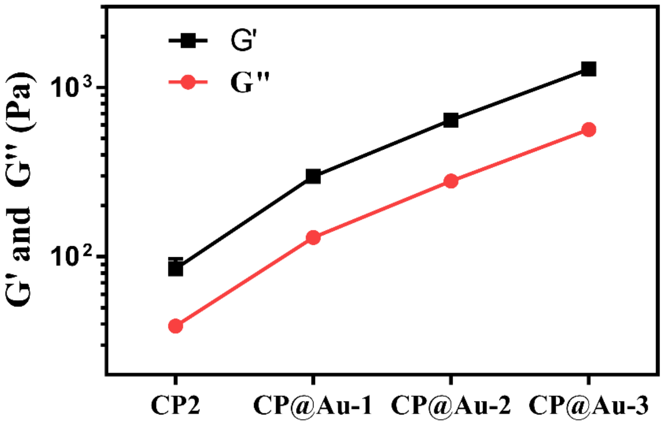

Figure S6. Rheological properties of the CP and CP@Au hydrogel, the G’and G” of hydrogels were improved with increasing GNRs content.

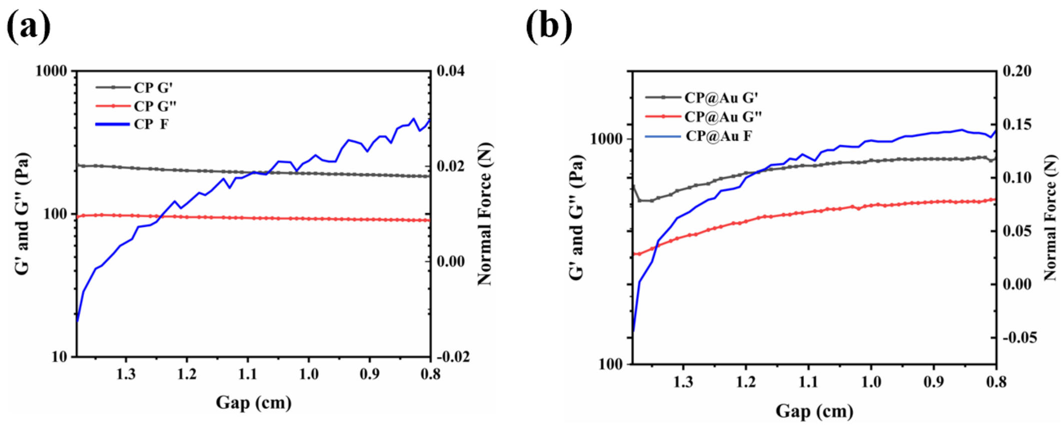

Figure S7. Rheological properties and normal force of the CP and CP@Au hydrogel with the gap change from 1.381 cm to 0.8 cm. The mechanical strength of CP was decreased and the mechanical strength of CP@Au hydrogel was increased while changing the gap distance. Moreover, the normal force of CP increased to 0.023 N and CP@Au increased to 0.15 N. According to the formula $P=F/S$ and the diameter of the plane was 25mm, the pressure of CP and CP@Au was 0.047 kPa and 0.31kPa, respectively. Due to the pressure of CP@Au was similar to the intraocular pressure (~1.3-3.0 kPa), the incorporation of GNRs into CP hydrogel was significantly important to resist intraocular pressure.

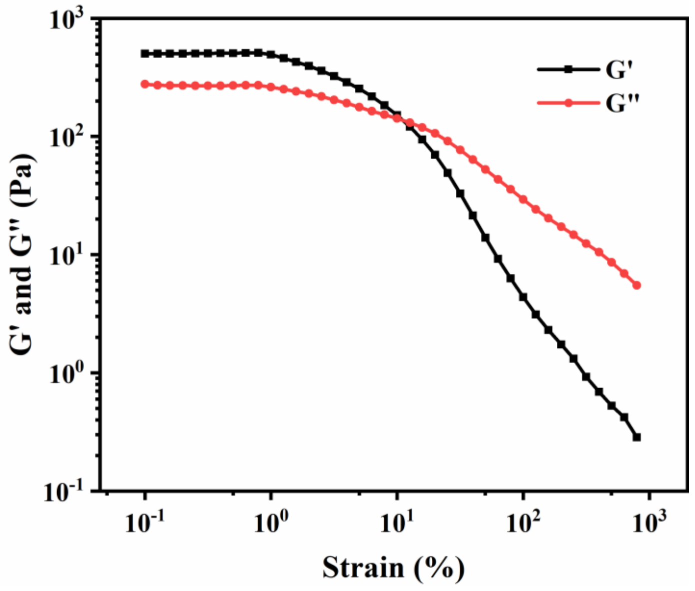

Figure S8. Strain-sweep measurements of CP@Au hydrogel over a range from 1% to 1000% strain. G’ and G” decrease with the increase of strain, indicating the shear-thinning properties of the CP@Au hydrogel. And the intersection points of G’and G” was 10% strain, the CP@Au hydrogel remained a statement of gel under a strain less than 10%, whereas hydergel became a sol state under more than 10% strain.

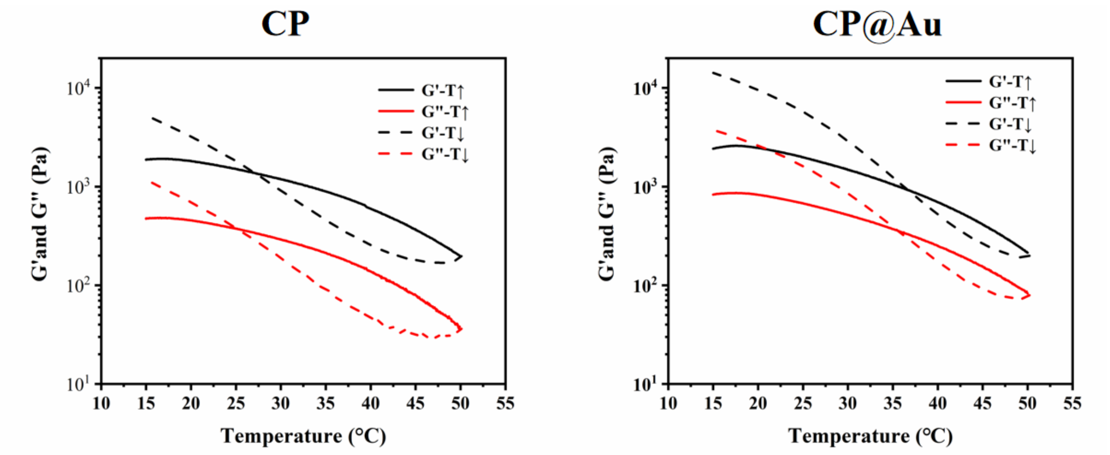

Figure S9. Dynamic temperature sweeps test of the CP and CP@Au hydrogel ranging from 15 to 50° C at a rate of 1° C min^{-1}.

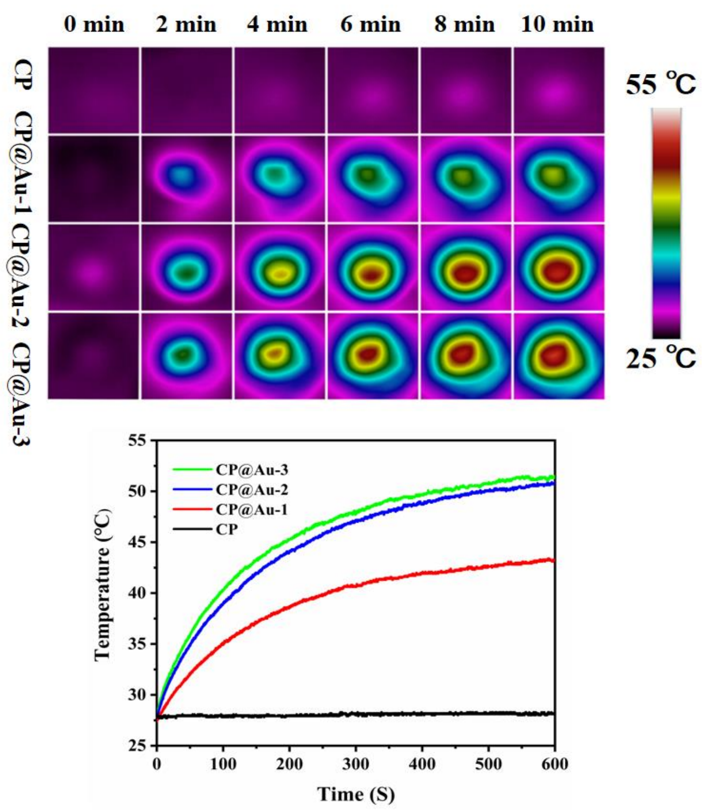

Figure S10. Infrared thermal images of the samples under 0.5 W/cm^2 laser irradiation and corresponding temperature change curves.

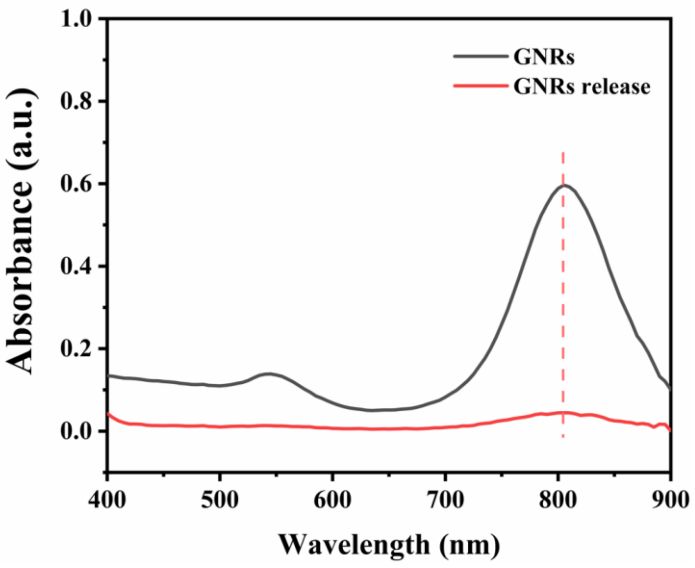

Figure S11. UV-vis absorption spectrum of the GNRs and GNRs released from CP@Au hydrogel after 5 min NIR laser irradiation. The absorption peaks of the samples were detected at 808 nm and the values were 0.59 and 0.045, indicating the minimum release amount of GNRs from CP@Au hydrogel.

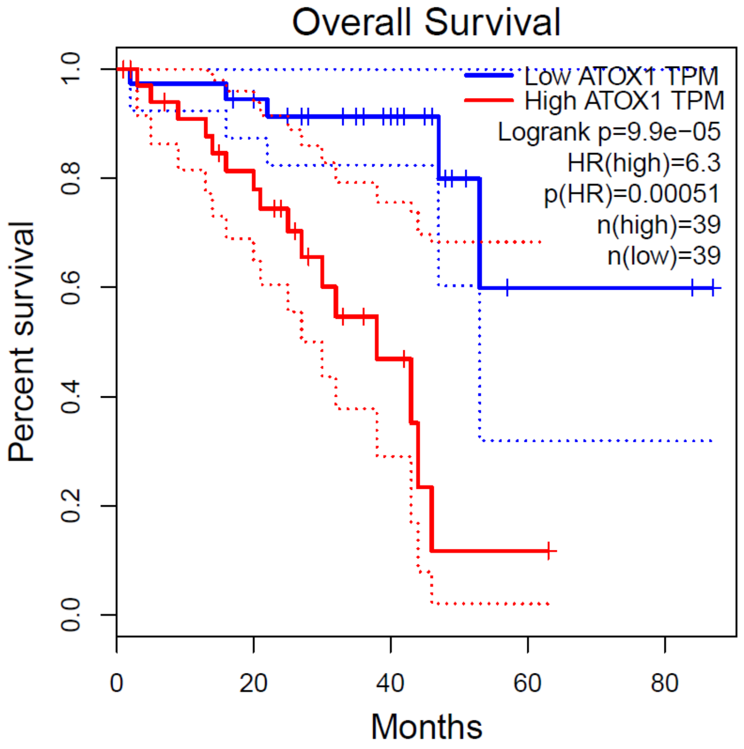

Figure S12. Kaplan-Meier survival analysis of melanoma patients in GEPIA.

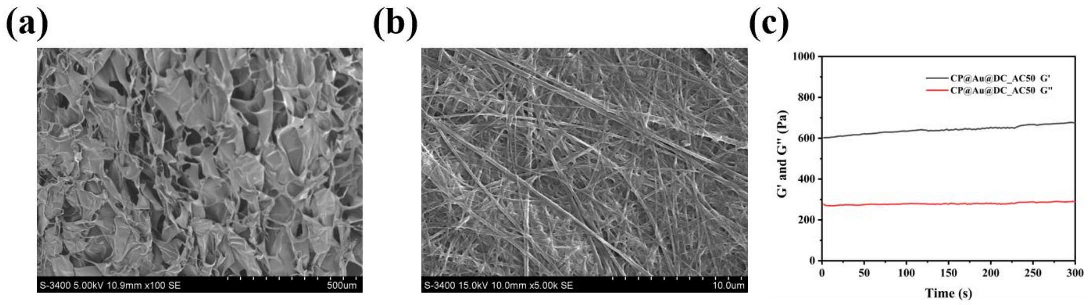

Figure S13. The properties of CP@Au@DC_AC50 hydrogel. SEM images of the porous structure (a), the nanofiber structure (b) and the Rheological property (c).

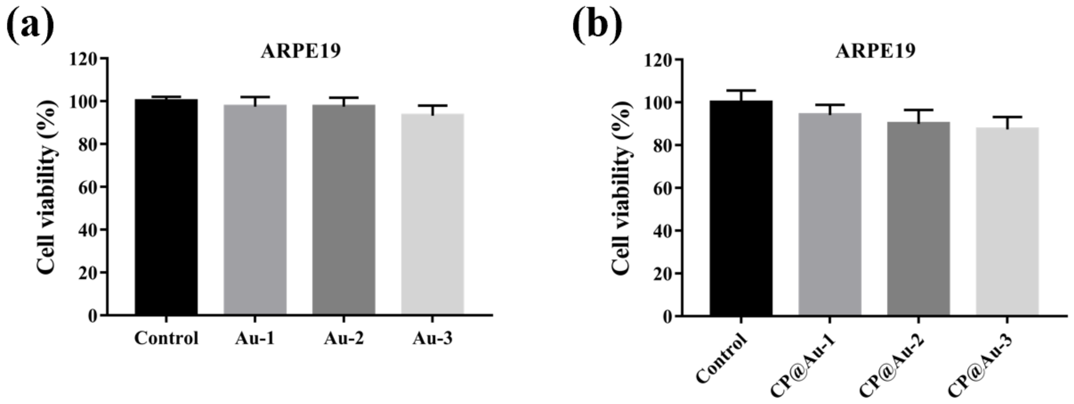

Figure S14. The viability of ARPE19 co-cultured with the GNRs (a) and CP@Au (b). The concentration of Au element in Au-1, Au-2 and Au-3 was 0.15 μM, 0.3 μM and 1.5 μM, respectively. The ratio of the CP@Au hydrogel and medium of CP@Au-1, CP@Au-2 and CP@Au-3 was 1:4, 1:2 and 1:1.

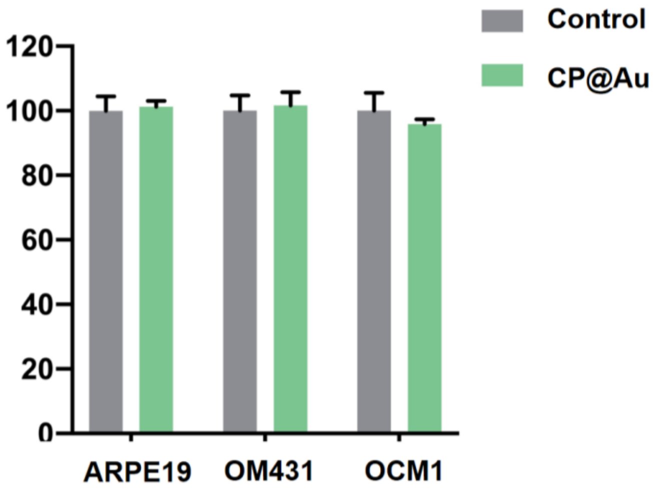

Figure S15. The viability of the cells (ARPE19, OM431 and OCM1) co-cultured with the CP@Au hydrogel over 48 h.

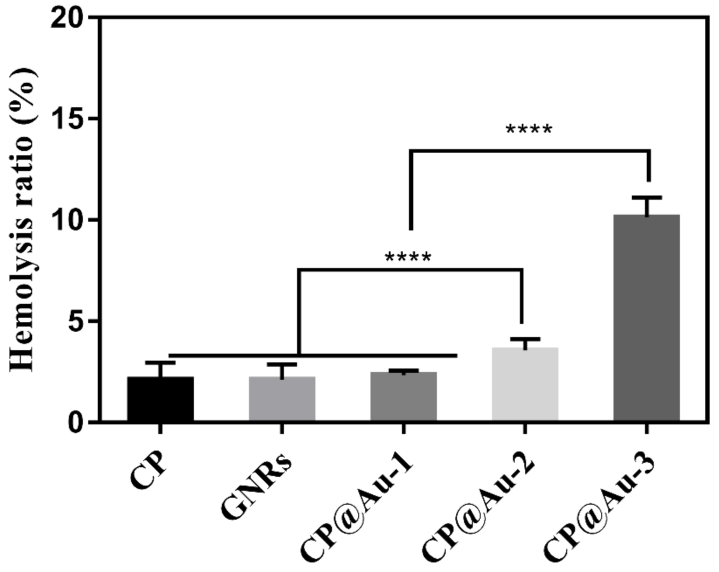

Figure S16. The hemolysis ratios of samples.

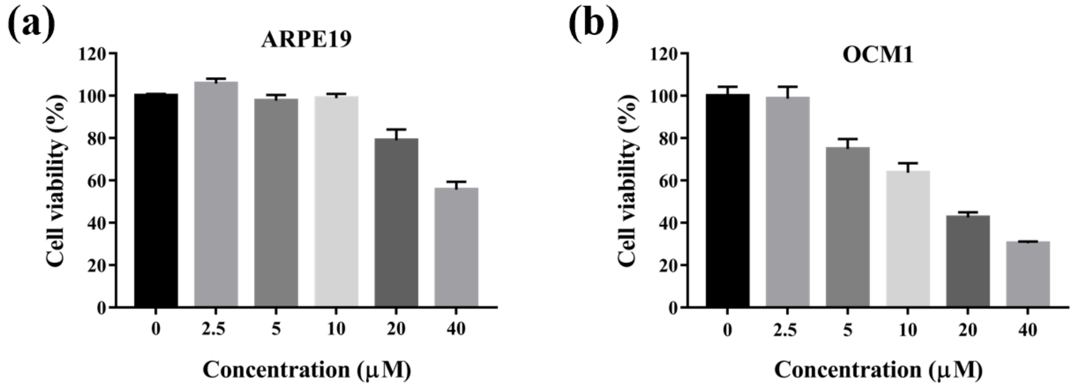

Figure S17. The viability of normal cell ARPE19 (a) and tumor cell OCM1 (b) co-cultured with DC_AC50 and the concentration of DC_AC50 was 0μM, 2.5 μM, 5μM, 10μM, 20μM and 40μM, respectively.

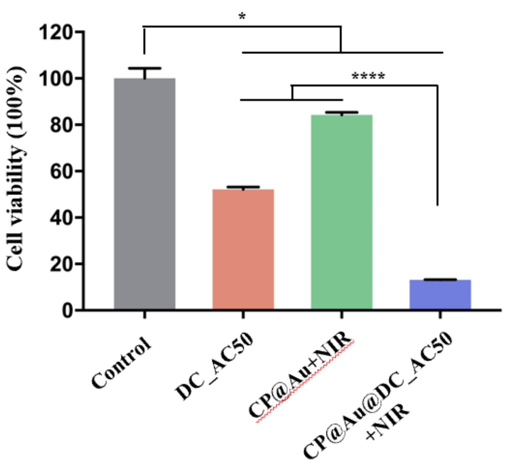

Figure S18. The viability of the OCM1 treated by different samples for 48 h.

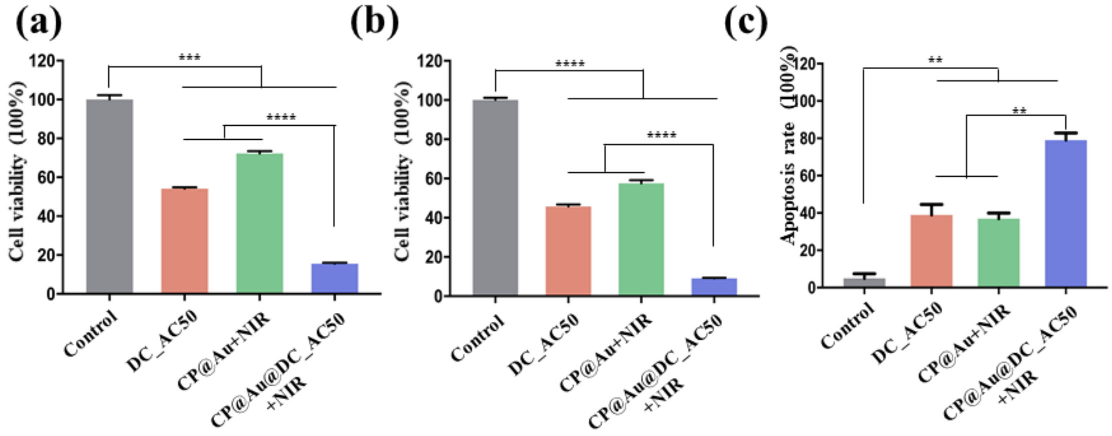

Figure S19. CCK8 assay of OM431 suffered by different treatments for 24 h (a) and 48 h (b). Apoptosis rate of OM431 cocultured with different samples over 24h (c).

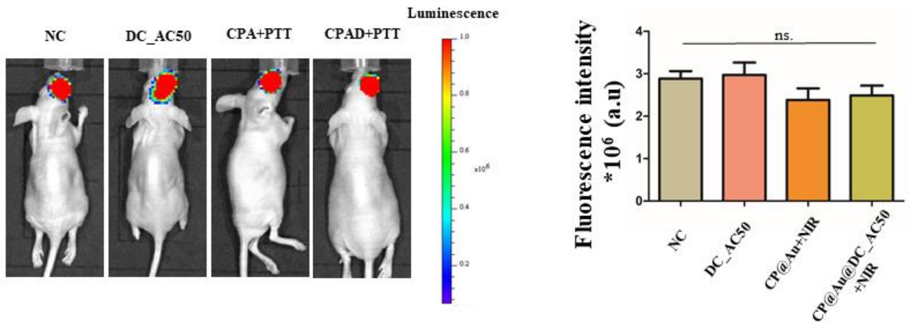

Figure S20. Animal fluorescence imaging of mice 5 days post OCM1/Luc cell injection and the corresponding statistical histogram of fluorescence intensity.

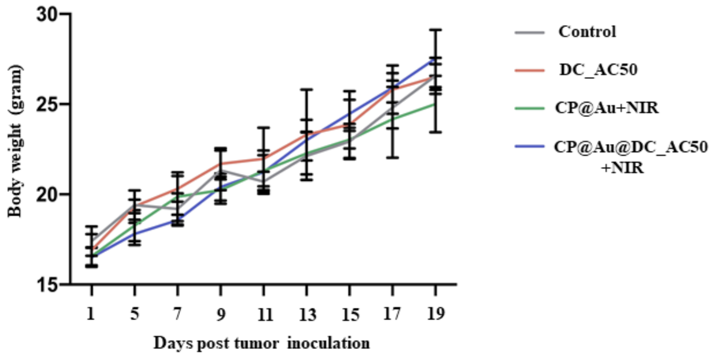

Figure S21. Body weight of the mice.
